# Modelling of SMA Vibration Systems in an AVA Example

**DOI:** 10.3390/ma14195905

**Published:** 2021-10-08

**Authors:** Waldemar Rączka, Jarosław Konieczny, Marek Sibielak

**Affiliations:** Department of Process Control, AGH University of Science and Technology, Al. Mickiewicza 30, 30-059 Krakow, Poland; koniejar@agh.edu.pl (J.K.); sibielak@agh.edu.pl (M.S.)

**Keywords:** modelling, vibration, shape memory alloys

## Abstract

Vibration suppression, as well as its generation, is a common subject of scientific investigations. More and more often, but still rarely, shape memory alloys (SMAs) are used in vibrating systems, despite the fact that SMA springs have many advantages. This is due to the difficulty of the mathematical description and the considerable effortfulness of analysing and synthesising vibrating systems. The article shows the analysis of vibrating systems in which spring elements made of SMAs are used. The modelling and analysis method of vibrating systems is shown in the example of a vibrating system with a dynamic vibration absorber (DVA), which uses springs made of a shape memory alloy. The formulated mathematical model of a 2-DOF system with a controlled spring, mounted in DVA suspension, uses the viscoelastic model of the SMA spring. For the object, a control system was synthesised. Finally, model tests with and without a controller were carried out. The characteristics of the vibrations’ transmissibility functions for both systems were determined. It was shown that the developed DVA can tune to frequency excitation changes of up to ±10%.

## 1. Introduction

Due to its advantages, SMAs are materials that are increasingly used in many areas of our life. They are widely used in engineering, as various types of actuators, connecting elements, clamps and springs, and in medicine to create, e.g., stents, occluders, artificial heart valves, dental burs and many others. There are many applications using all effects that occur in SMAs (one-way, two-way shape memory or pseudoelasticity) in medicine, aerospace and general engineering. In this paper, we focus on SMA applications in vibrating systems and problems with their modelling. As these are materials with complex temperature- and stress-induced phase transformations depending on many factors, their usage is preceded by more or less labourious calculations using various types of mathematical models. Depending on the phenomenon we want to analyse, macroscopic, mesoscopic or microscopic models are used. Khandelwal and Buravalla in [1] made a valued review of various types of models developed, among others, by Birman [2], Bernardini and Pence in [3] and Paiva and Savi [4], Smith [5] and Lagoudas [6], Achenbach [7], Müller [8], Seelecke [9,10,11] and many others. They mainly focused on continuum models to describe phenomena that occur in SMAs. The second group of models describing SMAs is input-output models describing SMAs as a black box. These models usually describe the hysteresis phenomenon that occurs in SMAs. Such models are useful when the macroscopic effects of phenomena occurring in SMAs are the thing we are interested in the most. There are two main models of this type: Preisach [12,13,14] and Duhem–Madelung models [5]. These models describe the hysteresis phenomenon and are applicative in the description of one-dimensional SMA objects with lumped parameters. Both of them are usually used to obtain the time responses of the SMA object. However, if we want to get the object’s response in the frequency domain, they are very labourious because each point of the chart should be determined separately. It is not easy to use these input-output models in frequency analyses, similar to the phenomenological models too. Thus, such analyses, due to their labour consumption, are rarely carried out; however, they are the fundamental tool in the design of vibrating systems such as vibration reduction systems or generators.

### Vibration Systems with SMA Modelling

The need of a SMA description in vibrating systems concerns many authors. The problem with the description of the vibrating system concerns authors in [15]. They describe the 1-DOF system with a SMA spring modelled by the constitutive model. They obtain time responses of the object. Moreover, Seelecke in [9] considers the 1-DOF with the constitutive model and obtained time courses as well as very valuable phase diagrams. The very interesting problem of energy dissipation in SMAs is described in work by [11]. Dissipative properties of SMAs can be applied in reduction systems. In this paper, the authors analysed the problem using a constitutive model as well. In all these works, the authors did not demonstrate frequency characteristics because they are difficult to obtain using constitutive models as mentioned above.

In the case of vibration reduction systems, their primary purpose is to minimise acceleration or displacement amplitudes [16,17,18,19,20]. Sometimes the goal is a reduction in monoharmonic vibrations. They can be caused by external excitation or result from object properties, structure, etc., such as natural frequency. Special active vibration reduction systems can mitigate such excitations. Sibielak et al. in [21,22,23] proposed one of the most interesting solutions. The authors developed a controller to reduce selected monoharmonic excitations to a requested level. Another well-known method is using DVA for the vibration mitigation of monoharmonic excitation. DVAs are designed for a single frequency of disturbance. The effectiveness of vibration reduction is most significant when damping in the absorber’s system is zero. In such a case, the vibration reduction bandwidth is the narrowest. When the frequency of disturbance changes, additional damping broadens the frequency bandwidth of the absorber’s operation instead of its effectiveness. Therefore, controlled absorbers are an alternative method to compensate for the influence of the changes in the frequency of disturbance vibrations. Such absorbers are adjusted to the actual disturbance frequency by modifying parameters such as the suspension spring stiffness, the damping coefficient or mass. Such a controlled DVA is named the adaptive vibration absorber (AVA).

Springs with controllable stiffness are constructed, e.g., from materials with a changeable Young’s modulus; often, this is SMA. The change in the stiffness of a SMA element is related to the modification of Young’s modulus caused by external factors such as heat or magnetic energy [24,25,26,27]. SMAs are materials in which a phase transition occurs, caused by supplied heat energy and/or external stress. Springs with a controllable stiffness coefficient are used in various vibration applications such as DVAs, AVAs [28,29], the resonant sieving screen [30] and others [31,32]. Generally, spring elements made of SMAs can be used in active, semi-active or passive systems [33]. Because active elements are made of SMAs and are characterised by considerable time constants amounting to 1 s or more, their use in active reduction systems is limited to very low-frequency vibrations. Therefore, they are more frequently used in semi-active, adaptive systems as elements with controllable parameters. In such cases, changes in their properties, such as stiffness and damping, could be relatively slow against frequency. In such a case, using springs with controlled stiffness in a DVA or AVA is an excellent idea, which is considered in few papers. For example, Williams et al. [34] used the SMA spring to control the suspension stiffness of an AVA. They built a physical model of such an absorber and performed its laboratory tests. They labouriously determined the frequency characteristics of the AVA for various temperatures, including the characteristics of the AVA with a "manually" tuned absorber. Then, in [35], Williams et al. formulated a mathematical model of the SMA spring absorber. In the model, SMA spring parameters are determined based on laboratory tests and tabulated. In the paper [36], Williams et al. proposed a controller for an AVA and performed its time characteristics. The authors presented the time characteristics because obtaining the frequency characteristics using constitutive models is very labour-intensive and therefore not used. This is the fundamental problem in designing and analysing vibration systems using SMAs, both those that generate vibrations or those that reduce them. The same problem occurs in the synthesis and analysis of control systems with actuators made of SMAs.

**Figure 1:**
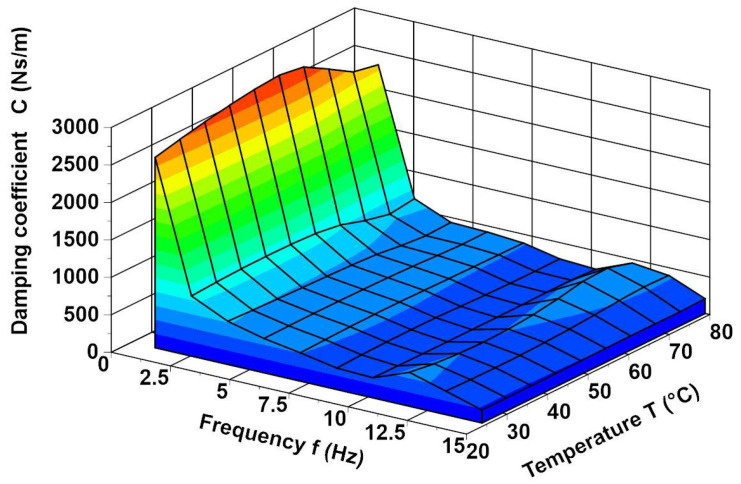
The damping of the spring as a function of frequency for selected temperatures.

**Figure 2:**
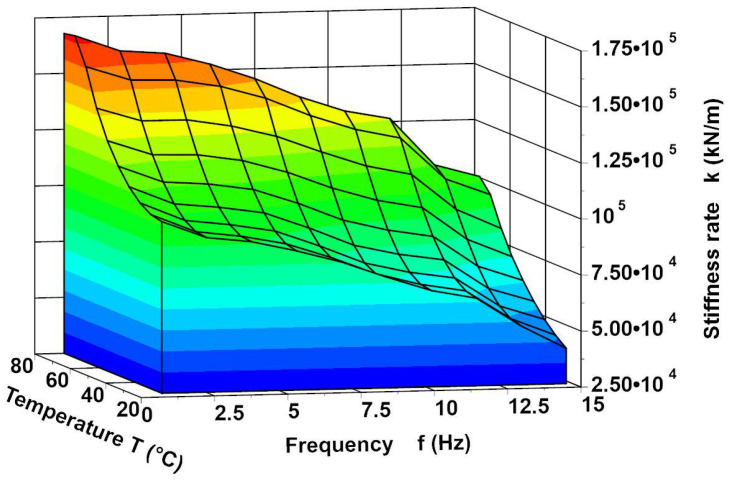
The spring rate k as a function frequency for selected temperatures.

The development of a SMA modelling method to facilitate the frequency analysis was the primary motivation behind developing the viscoelastic SMA model described in [27]. The model of the SMA spring was formulated on the basis of the analysis of the spring static characteristics [27]. It was observed that such a spring has significant damping. Hence, it was hypothesised that the spring reaction force can be described by the Formula (1). The spring is made of NiTi (Ni 48%, Ti 46%, Cu 6% and C 0.05%). The characteristic temperatures of the alloy are $M_{s}=45\;{}^{\circ}C,M_{f}=30\;{}^{\circ}C,\;A_{s}=50\;{}^{\circ}C,\;A_{f}=70\;{}^{\circ}C$. The spring tests showed that its characteristic not only depends on the temperature but also varies depending on the frequency of excitation [27]. It turned out that the coefficients k and c of the spring depend not only on the temperature but also on the frequency, which can be seen in Figure 1 and Figure 2. These figures show the values of the spring coefficients k and c as a function of the frequency for selected temperatures. We can see that with a higher frequency, the stiffness rate and damping coefficients decrease. This phenomenon is examined by Piedboeuf et al. [37], Guher et al. [38] and Karakalas et al. [39] too. Formula (1) is a SMA spring model described in [27]. The model is written in the form (2) after taking into account (3) and (4). The values of the determination methods of the coefficients and the approximating functions (3) and (4) are presented in the article, [27].

(1) $$F=kz+c\dot{z}$$

where:

(2) $$F=k\left(T,\omega \right)z+c\left(\omega \right)\dot{z}$$

where:

k(T,ω)—SMA spring stiffness rate function,

c(ω)—SMA spring damping function,

F—SMA spring reaction force,

z—SMA spring deflection,

T—SMA spring temperature,

ω—frequency of excitation.

The stiffness rate function k(T,ω) is explained using the following formula:

(3) $$k\left(T,\omega \right)=a_{1}+a_{2}\omega +a_{3}\omega^{2}+a_{4}T$$

where the coefficients $a_{1}$, $a_{2}$ and $a_{3}$ are determined using the least-squares method and equal: $a_{1}=70,952,\;a_{2}=-213.01,\;a_{3}=-5.214,\;a_{4}=1148.8$.

The damping function c(ω) is approximated using the following formula.

(4) $$c\left(\omega \right)=b_{1}+b_{2}\frac{1}{\omega }$$

where the coefficients $b_{1},\;b_{2},\;b_{3}$ and $b_{4}$ are determined using the least-squares method and equal: $b_{1}=-1.91,\;b_{2}=17,100$.

This viscoelastic model of SMAs, widely described in the article [27], enables the frequency analysis of vibrating systems with spring elements made of SMAs.

In this paper, the use of the model is shown in the example of the controlled AVA vibration absorber. Since the viscoelastic model of the SMA spring was used to formulate the mathematical model of the absorber, it was possible to perform a frequency analysis of both the passive and active systems. The results of the system tests are presented below in a graphic form. An AVA with a controlled spring made of a SMA was proposed due to the fact that the properties of the SMA spring can be controlled by controlling only its temperature. Thanks to this, the resonant frequency of the absorber can be easily controlled.

## 2. Materials and Methods

**Figure 3:**
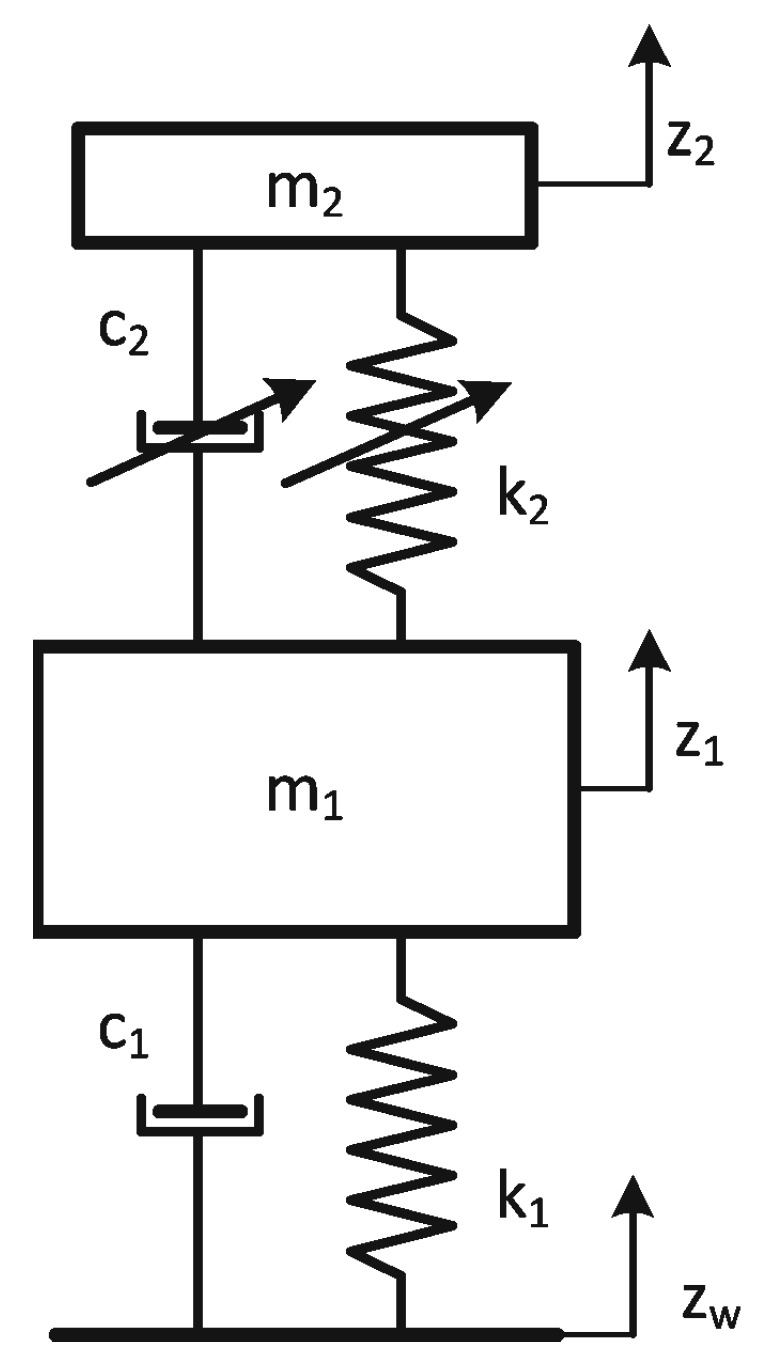
Calculation diagram of the vibration reduction system.

The mathematical model (5), (6) of the AVA with a controlled dynamic damper was formulated based on its diagram shown in Figure 3. The absorber in the form of a mass $m_{2}=12\;\mathrm{kg}$ is suspended by a SMA spring and protects the main mass $m_{1}=25\;\mathrm{kg}$. The SMA spring is represented by two elements, a controlled spring $k_{2}$ and a controlled damper $c_{2}$ connected in parallel. The protected mass $m_{1}$ is excited by the kinematic excitation $z_{w}=Asin\;\left(\omega t\right)$. For the sake of the notation simplification, the symbols $z_{1}=z_{1}\left(t\right)$ and $Z_{1}=Z_{1}\left(s\right)$ were adopted.

(5) $$\begin{array}{c} k_{1}\left(z_{w}-z_{1}\right)+c_{1}\left({\dot{z}}_{w}-{\dot{z}}_{1}\right)=m_{1}{\ddot{z}}_{1}+k_{2}\left(z_{1}-z_{2}\right)+c_{2}\left({\dot{z}}_{1}-{\dot{z}}_{2}\right) \end{array}$$

(6) $$\begin{array}{c} k_{2}\left(z_{1}-z_{2}\right)+c_{2}\left({\dot{z}}_{1}-{\dot{z}}_{2}\right)=m_{2}{\ddot{z}}_{2} \end{array}$$

The SMA spring was described using a viscoelastic model with variable parameters (2), (3), (4) and is described above. After a Laplace transformation of the system of Equations (5) and (6), we obtained:

(7) $$\begin{array}{c} k_{1}\left(Z_{w}-Z_{1}\right)+c_{1}\left(Z_{w}-Z_{1}\right)s=m_{1}Z_{1}s^{2}+k_{2}\left(Z_{1}-Z_{2}\right)+c_{2}\left(Z_{1}-Z_{2}\right)s \end{array}$$

(8) $$\begin{array}{c} k_{2}\left(Z_{1}-Z_{2}\right)+c_{2}\left(Z_{1}-Z_{2}\right)s=m_{2}Z_{2}s^{2} \end{array}$$

Equations (7) and (8) were written in matrix form:

(9) Ax=Bu

where:

(10) $$A=\left[\begin{array}{cc} \left(-m_{1}s^{2}-c_{1}s-k_{1}-k_{2}-c_{2}s\right) & \left(k_{2}+c_{2}s\right)\\ \left(k_{2}+c_{2}s\right) & -\left(m_{2}s^{2}+c_{2}s+k_{2}\right) \end{array}\right]$$

(11) $$B=\left[\begin{array}{c} -\left(k_{1}+sc_{1}\right)\\ 0 \end{array}\right]$$

(12) $$x=\left[\begin{array}{c} z_{1}\\ z_{2} \end{array}\right]$$

(13) $$u=z_{w}$$

To solve the system of Equation (9), we calculate determinants. The Formula (14) gives the principal determinant of A:

(14) $$det\left(A\right)=\left(m_{1}s^{2}+c_{1}s+k_{1}+k_{2}+c_{2}s\right)\left(m_{2}s^{2}+c_{2}s+k_{2}\right)-{\left(k_{2}+c_{2}s\right)}^{2}$$

Determinant $A_{z1}$ is written in the form:

(15) $$det\left(A_{z1}\right)=z_{w}\left(k_{1}+c_{1}s\right)\left(m_{2}s^{2}+c_{2}s+k_{2}\right)$$

Determinant $A_{z2}$ is written in the form:

(16) $$det\left(A_{z2}\right)=z_{w}\left(k_{1}+c_{1}s\right)\left(k_{2}+c_{2}s\right)$$

The transfer function $G_{z1zw}$ for input $z_{w}$ and output $z_{1}$ is:

(17) $$G_{z1zw}\left(s\right)=\frac{\left(k_{1}+c_{1}s\right)\left(m_{2}s^{2}+c_{2}s+k_{2}\right)}{\left(m_{1}s^{2}+c_{1}s+k_{1}+k_{2}+c_{2}s\right)\left(m_{2}s^{2}+c_{2}s+k_{2}\right)-{\left(k_{2}+c_{2}s\right)}^{2}}$$

The transfer function $G_{z2zw}$ for input $z_{w}$ and output $z_{2}$ is:

(18) $$G_{z2zw}\left(s\right)=\frac{\left(k_{1}+c_{1}s\right)\left(k_{2}+c_{2}s\right)}{\left(m_{1}s^{2}+c_{1}s+k_{1}+k_{2}+c_{2}s\right)\left(m_{2}s^{2}+c_{2}s+k_{2}\right)-{\left(k_{2}+c_{2}s\right)}^{2}}$$

The transfer function $G_{z2z1}$ for input $z_{1}$ and output $z_{2}$ is:

(19) $$G_{z2z1}\left(s\right)=\frac{\left(k_{2}+c_{2}s\right)}{\left(m_{2}s^{2}+c_{2}s+k_{2}\right)}$$

Thus, the spectral transmittances of such an object for input displacement $z_{w}$ and output displacements $z_{1}$ and $z_{2}$ are given by Equations (20) and (21), respectively.

(20) $$\begin{array}{c} G_{z1zw}\left(j\omega \right)=\frac{-m_{2}c_{1}j\omega^{3}-P_{1}\omega^{2}+P_{2}j\omega +k_{1}k_{2}}{m_{1}m_{2}\omega^{4}-\left(m_{2}c_{1}+Mc_{2}\right)j\omega^{3}-\left(\;P_{1}+Mk_{2}\right)\omega^{2}+P_{2}j\omega +k_{1}k_{2}} \end{array}$$

(21) $$G_{z2zw}\left(j\omega \right)=\frac{-c_{1}c_{2}\omega^{2}+P_{2}j\omega +k_{1}k_{2}}{m_{1}m_{2}\omega^{4}-\left(m_{2}c_{1}+Mc_{2}\right)j\omega^{3}-\left(\;P_{1}+Mk_{2}\right)\omega^{2}+P_{2}j\omega +k_{1}k_{2}}$$

where:

$M=m_{2}+m_{1}$,

$P_{1}=m_{2}k_{1}+c_{1}c_{2}$,

$P_{2}=c_{1}k_{2}+k_{1}c_{2}$.

The spectral transmittance of the absorber for the input $z_{1}$ protected the mass displacement, and the output $z_{2}$ damper displacement is given by the Equation (22).

(22) $$G_{z2z1}\left(j\omega \right)=\frac{\left(c_{2}j\omega +k_{2}\right)}{\left(-m_{2}\omega^{2}+c_{2}j\omega +k_{2}\right)}$$

## 3. Results

**Figure 4:**
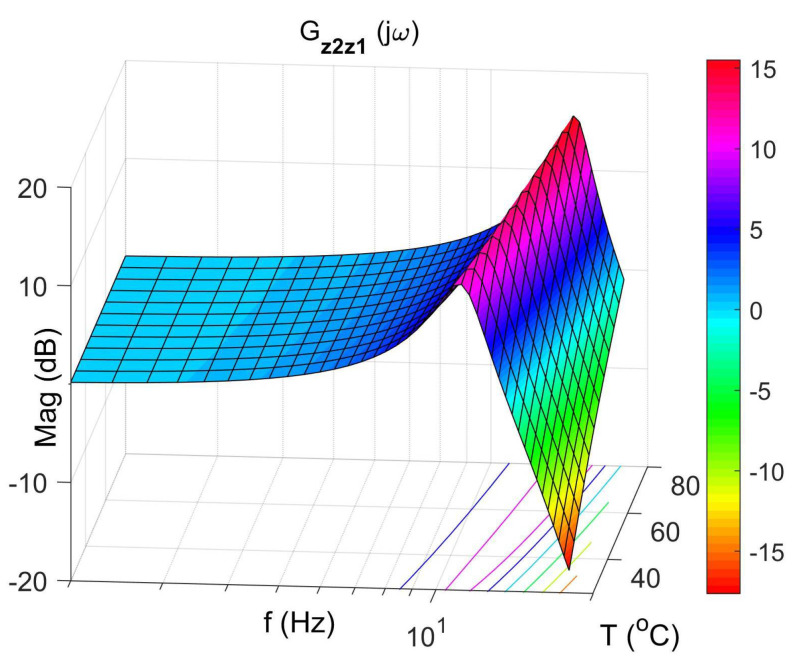
Vibration transmissibility function of the absorber as a function of frequency and temperature, the transfer function $G_{z2z1}$.

**Figure 5:**
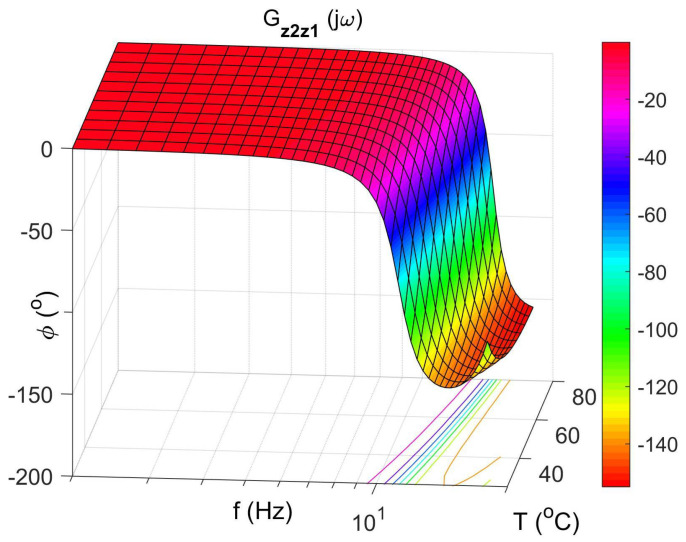
Absorber phase shift as a function of frequency and temperature, the transfer function $G_{z2z1}$.

**Figure 6:**
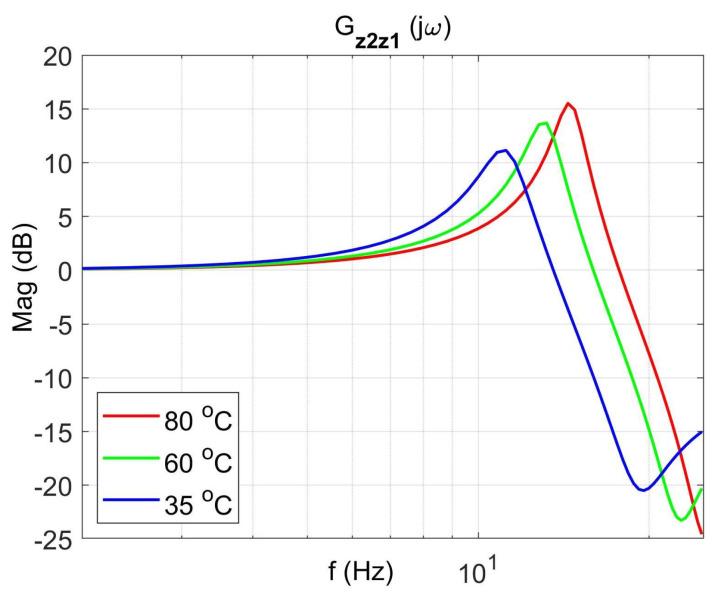
Vibration transmissibility functions of the absorber as a function of frequency for selected temperatures 25 °C, 60 °C, 80 °C, the transfer function $G_{z2z1}$.

**Figure 7:**
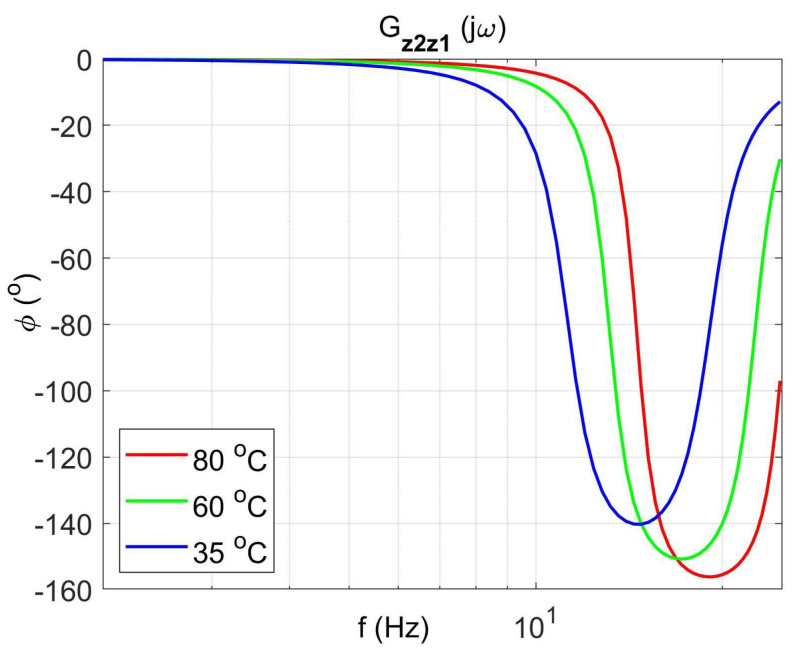
The absorber phase shifts as a function of frequency for selected temperatures 25 °C, 60 °C, 80 °C, the transfer function $G_{z2z1}$.

Figure 4 shows the vibration transmissibility function of the absorber with the mass $m_{2}$ described by the transfer function (22) as a function of the frequency of the displacement signal $z_{1}$ and the temperature of the SMA spring. The resonance frequency of the absorber increases with an increasing temperature from 11.1 Hz in the temperature 25 °C up to 14.5 Hz in the temperature 80 °C. It is a result of SMA spring features described by Equations (3) and (4). Thus, by controlling the spring temperature, we control the resonant frequency of the absorber in the range 11.1 Hz to 14.5 Hz. This means that by changing only the temperature of the SMA spring, we can tune the absorber to the frequency of disturbance $z_{w}$. Then, Figure 5 shows the phase shift between the displacements $z_{1}$ and $z_{2}$ for the absorber. Figure 6 and Figure 7 show the vibration transmissibility functions and phase shifts of the absorber for selected temperatures (25 °C, 60 °C, 80 °C).

**Figure 8:**
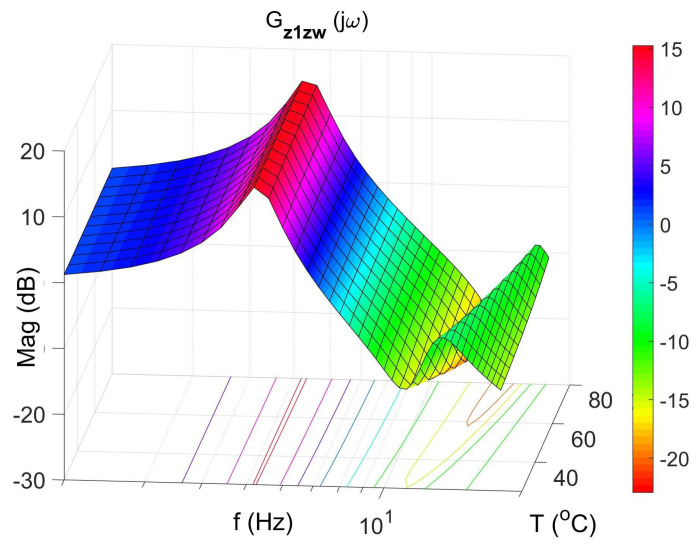
Vibration transmissibility function of disturbance $z_{w}$ to the protected mass $m_{1}$ as a function of frequency and temperature, the transfer function $G_{z1zw}$.

**Figure 9:**
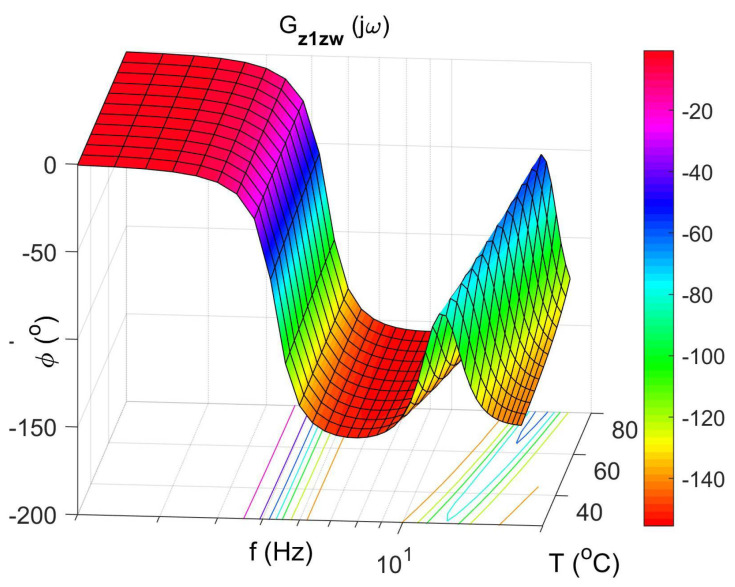
The phase shift of protected mass as a function of frequency and temperature, the transfer function $G_{z1zw}$.

**Figure 10:**
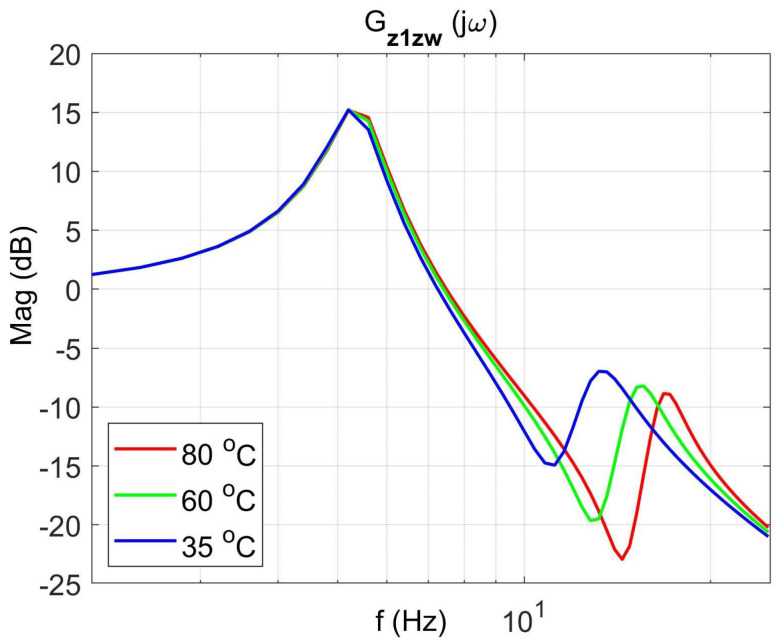
Vibration transmissibility functions of disturbance $z_{w}$ to the protected mass $m_{1}$ as a function of frequency for selected temperatures 25 °C, 60 °C, 80 °C, the transfer function $G_{z1zw}$.

**Figure 11:**
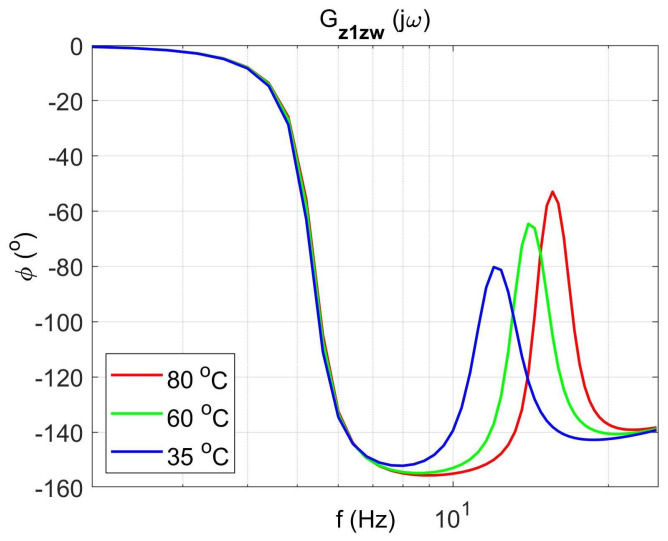
Absorber phase shift as a function of frequency for selected temperatures 25 °C, 60 °C, 80 °C, the transfer function $G_{z2zw}$.

**Figure 12:**
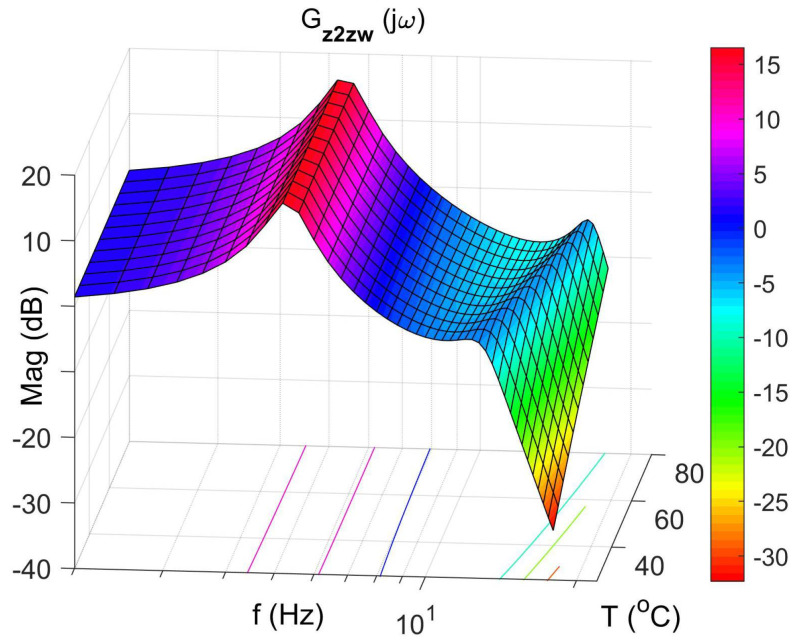
Vibration transmissibility function of disturbance $z_{w}$ to the absorber mass $m_{2}$ as a function of frequency and temperature, the transfer function $G_{z2zw}$.

**Figure 13:**
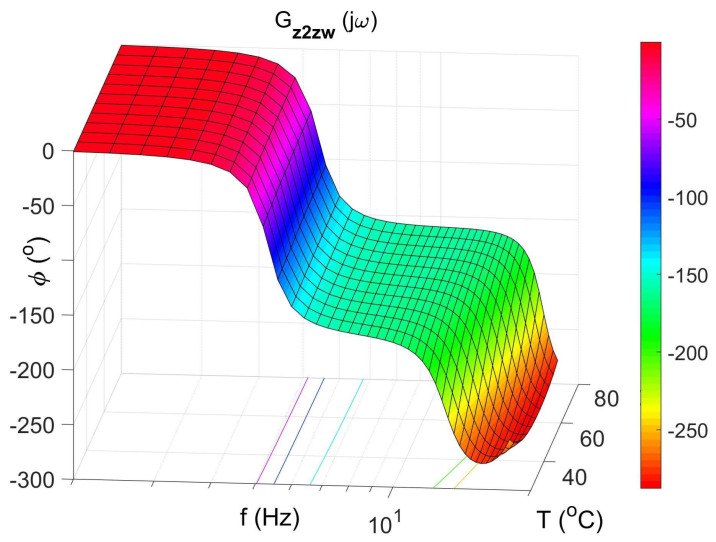
Protect mass phase shift between displacements $z_{w}$ and $z_{2}$ as a function of frequency and temperature, the transfer function $G_{z2zw}$.

**Figure 14:**
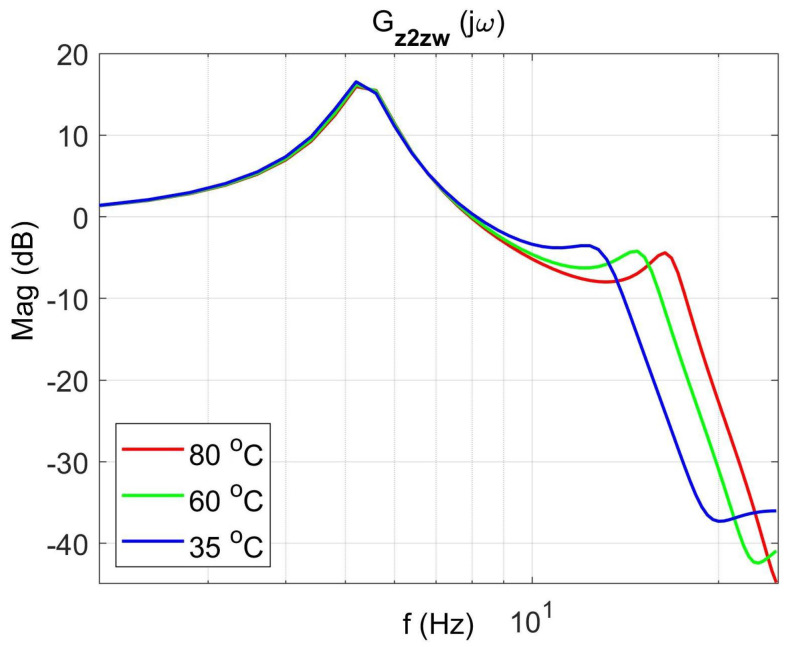
Vibration transmissibility functions of disturbance $z_{w}$ to the absorber mass $m_{2}$ as a function of frequency for selected temperatures 25 °C, 60 °C, 80 °C, the transfer function $G_{z2zw}$.

**Figure 15:**
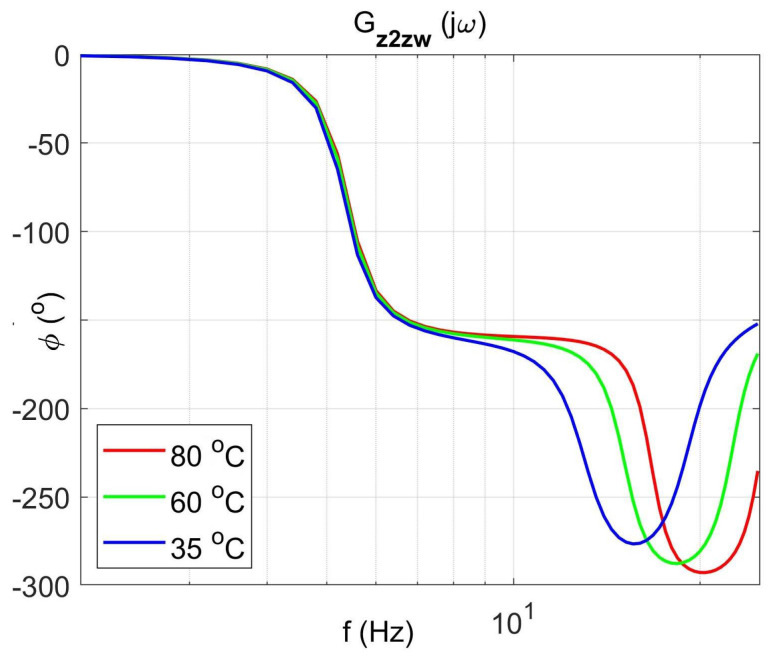
Absorber phase shift functions between displacements $z_{w}$ and $z_{2}$ as a function of frequency for selected temperatures 25 °C, 60 °C, 80 °C, the transfer function $G_{z2zw}$.

Figure 8, Figure 9, Figure 10 and Figure 11 show similar graphs for the protected mass $m_{1}$. In turn, Figure 12, Figure 13, Figure 14 and Figure 15 show the vibration transmissibility functions and the phase shifts between the displacements $z_{w}$ and $z_{2}$ for the entire 2-DOF system described by the transmittance $G_{z2zw}$.

**Figure 16:**
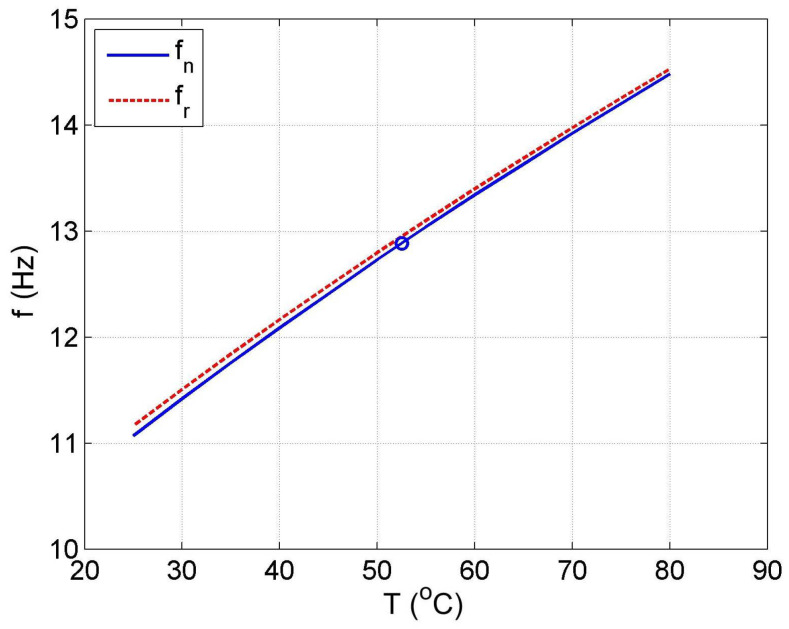
Chart of the natural frequency $f_{n}$ (solid line) and the resonance frequency $f_{r}$ (dashed line).

Figure 12 shows the vibration transmissibility function of the system described by the transfer function (21), with the input $z_{w}$, the displacement of excitation and the output $z_{2}$ and the displacement of the mass of the absorber $m_{2}$. Figure 12 shows the significant change in the resonant frequency of the absorber due to the change in the stiffness and damping of the SMA spring caused by the change in its temperature. Then, Figure 16 shows the change in the natural frequency of the absorber (solid line) as a function of the spring temperature within the allowable range. The value of the natural frequency $f_{n}$ of the dynamic damper can be calculated from the Formula (23). For comparison, in the figure, the change in the resonance frequency $f_{r}$ of the absorber as a function of the temperature is shown too.

(23) $$f_{n}=2\pi \frac{a_{2}+\sqrt{a_{2}{}^{2}+4\left(m-a_{3}\right)\left(a_{1}+a_{4}T\right)}}{2\left(m-a_{3}\right)}$$

**Figure 17:**
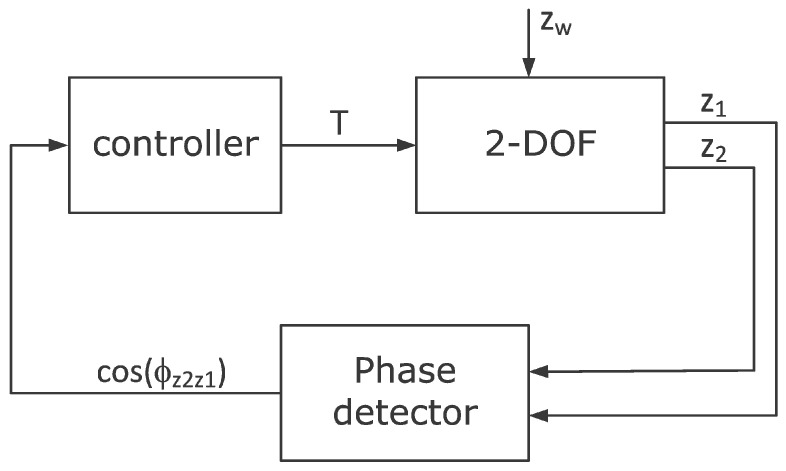
Control system block scheme.

The ability to change the resonant frequency of the absorber enables it to be adjusted to the changing frequency of the disturbance $z_{w}$. Such tuning is rational if it is performed automatically. For this purpose, the control system shown in Figure 17 was proposed. The goal of the control system is to adjust the resonant frequency of the absorber to the disturbance frequency $z_{w}$. In this case, the control system uses the fact that the resonant frequency of the absorber can be estimated with the natural frequency, and the phase shift $\varphi_{z2z1}$ between the displacement $z_{1}$ and the displacement $z_{2}$ is −90°. For this reason, it was decided that the feedback signal would be the cosine of the phase shift angle $\varphi_{z2z1}$. This signal is estimated in the "phase detector" block based on the Formula (25). In this case, the estimation error for the observation time $T_{o}$ being multiple periods of forced oscillation is equal to zero. In general, the estimation error of the estimate is always inversely proportional to the observation time $T_{o}$.

In this example, it was assumed that the nominal natural frequency of the absorber is $f_{no}=12.9\;\mathrm{Hz}$, and is equal to the nominal frequency of disturbance $z_{w}$. A circle in Figure 16 marks this value. This frequency is obtained in the SMA spring temperature $T_{n}=52.5$ °C in the considered AVA. In this case, for an operating spring temperature range of 25 °C to 80 °C, the damper operating frequency range is 11.1 Hz to 14.5 Hz. The natural frequency can therefore be varied by more than ±10% from its nominal value $f_{no}$.

After the above consideration, the nonlinear controller was proposed in the form:

(24) $$T=sat(K\cdot cos\left(\varphi_{z2z1}\right))$$

where:

(25) $$sat\left(v\right)=\{\begin{array}{c} 80\;for\;v>80\;\\ v\\ 25\;for\;v<25 \end{array}$$

(26) $$cos\left(\varphi_{z2z1}\right)=\frac{\frac{1}{T_{o}}\int_{t-T_{o}}^{t}z_{w}\left(\tau \right)z_{2}\left(\tau \right)d\tau }{\sqrt{\frac{1}{T_{o}}\int_{t-T_{o}}^{t}z_{2}^{2}\left(\tau \right)d\tau }\sqrt{\frac{1}{T_{o}}\int_{t-T_{o}}^{t}z_{1}^{2}\left(\tau \right)d\tau }}$$

Equation (26) follows directly from the definition of the dot product (27) between vectors in the functional space $L^{2}\left(\left[0,T\right],R\right)$.

(27) $$\langle v,w\rangle =\frac{1}{T}\int_{0}^{T}v\left(\tau \right)w\left(\tau \right)d\tau$$

The norm in the vector space $L^{2}\left(\left[0,T\right],R\right)$ generated by this dot product is expressed by the following formula:

(28) $$||v||=\sqrt{\langle v,v\rangle }$$

In this case, the cosine of the angle φ between the two vectors *v* and *w* is expressed as follows:

(29) $$cos\left(\varphi \right)=\frac{\langle v,w\rangle }{||v||\cdot ||w||}$$

If vectors v and w are harmonic functions of the same frequency, and the time *T* is a multiple of the period, then the angle φ corresponds to the phase shift angle between these functions.

Spectral transmittances of the 2-DOF with the controller are given by Formulas (20)–(22) and (30), (31) formulas describing the SMA spring.

(30) $$k_{2}\left(T,\omega \right)=a_{1}+a_{2}\omega +a_{3}\omega^{2}+a_{4}sat(Kcos\left(\varphi_{z2z1}\right))$$

(31) $$c_{2}\left(\omega \right)=b_{1}+b_{2}\frac{1}{\omega }$$

**Figure 18:**
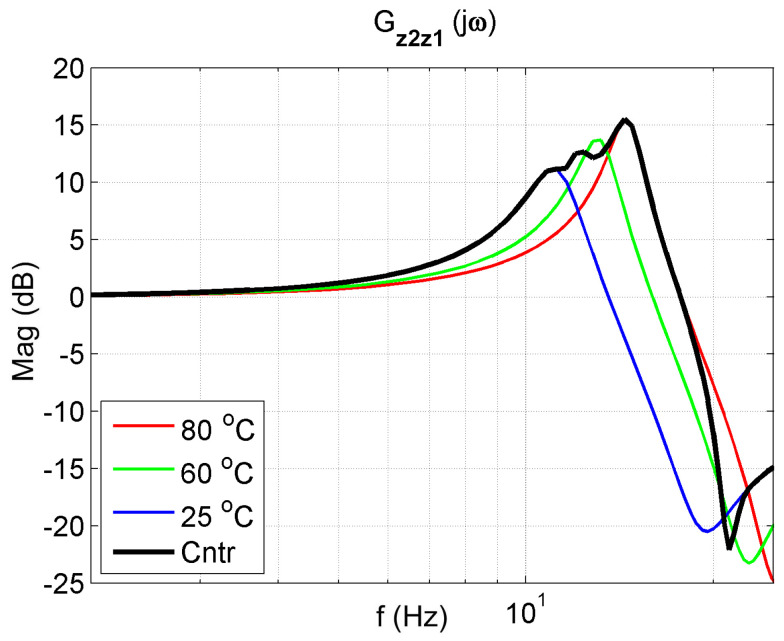
Vibration transmissibility functions of the passive absorber for selected temperatures of 25 °C, 60 °C, 80 °C and the controlled absorber (black). The transfer function $G_{z2z1}$ describes the object.

**Figure 19:**
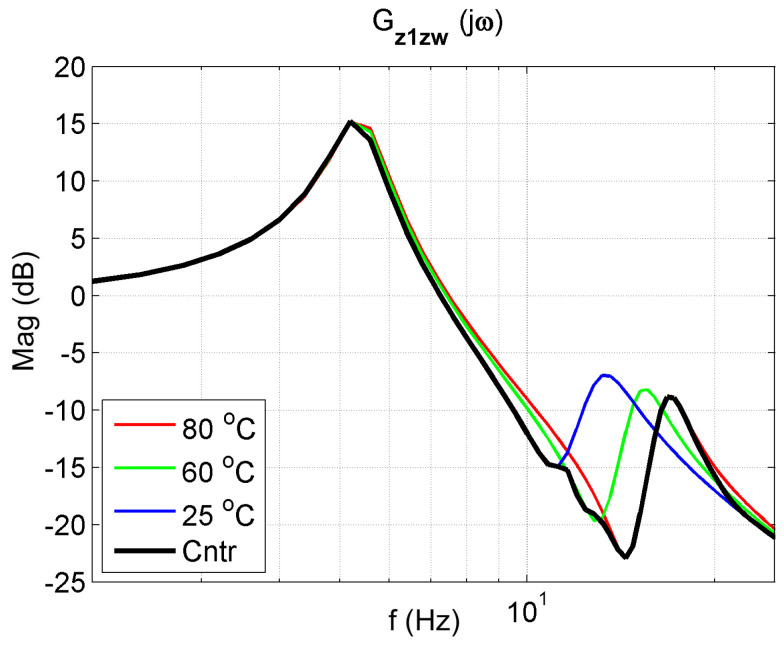
Vibration transmissibility functions of disturbance $z_{w}$ to the protected mass $m_{1}$ of the passive absorber for selected temperatures of 25 °C, 60 °C, 80 °C and the controlled absorber (black). The transfer function $G_{z1zw}$ describes the object.

**Figure 20:**
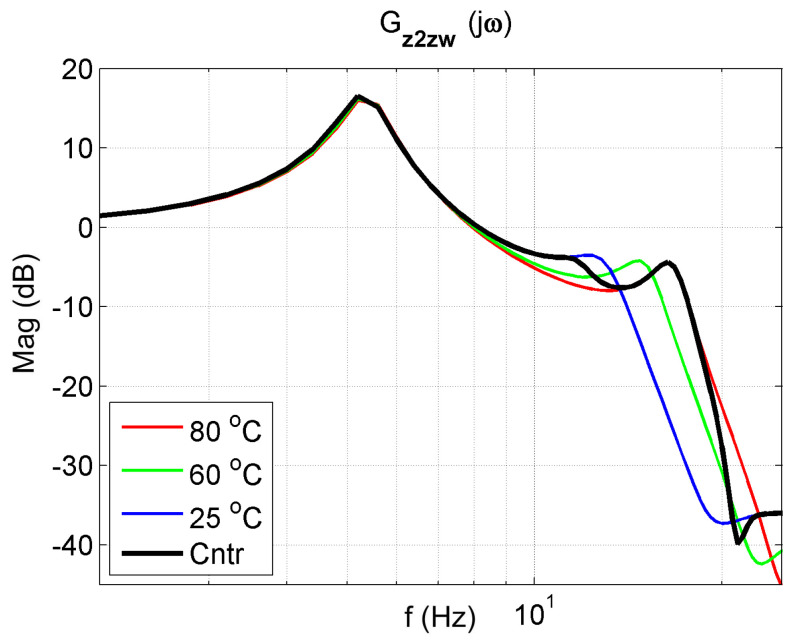
Vibration transmissibility functions of disturbance $z_{w}$ to the mass $m_{2}$ of the passive absorber for selected temperatures of 25 °C, 60 °C, 80 °C and the controlled absorber (black). The transfer function $G_{z2zw}$ describes the object.

The frequency response functions of the 2-DOF system with the controller for K = 50 are presented in Figure 18, Figure 19 and Figure 20. The frequency characteristics of the closed system in the figures are marked in black. An analysis of these figures shows that the control system protects the mass $m_{1}$ the best. The reduction in mass $m_{1}$ vibrations ($z_{1}$) is better in a broader range than in controlled systems. Thanks to changing its parameters, the controlled system can tune to the disturbance and, therefore, reduce vibration better.

## 4. Conclusions

The paper presents the developed mathematical model of the AVA system using a viscoelastic model of the SMA spring. The developed mathematical model enables numerical simulations in the frequency domains. The AVA was only an example of the application of this viscous model because, thanks to the use of the SMA spring, the AVA system can be tuned in real-time to the changing frequency of the disturbance in order to minimise the vibrations of the protected mass. As one can see, the viscous model enables the effective analysis of vibrating systems equipped with the SMA spring elements. Its characteristics can be easily determined using standard analytical methods. The results of the simulation tests of the model in the form of frequency characteristics are easy to obtain. Therefore, the work on the synthesis of the system is practical.

Additionally, it was shown that control systems can also be efficiently synthesised. For this purpose, a nonlinear control system was proposed and modelled. The tests of the AVA working in open and closed systems showed that thanks to the use of a controlled SMA spring, it is possible to perform an AVA which adjusts itself to the disturbance frequency on an ongoing basis, and it is better than passive systems because it operates in the broader frequency range.

## Data Availability

The data presented in this study are available on request from the corresponding author.
